# HMGB1 cytoplasmic translocation in patients with acute liver failure

**DOI:** 10.1186/1471-230X-11-21

**Published:** 2011-03-15

**Authors:** Rong-Rong Zhou, Shu-Shan Zhao, Ming-Xiang Zou, Pan Zhang, Bao-Xing Zhang, Xia-Hong Dai, Ning Li, Hong-Bo Liu, Haichao Wang, Xue-Gong Fan

**Affiliations:** 1 Department of Infectious Diseases, Xiangya Hospital, Central South University, Changsha, 410008, Hunan, PR China; 2 Eight-year Program, Central South University, Changsha, 410008, Hunan, PR China; 3 Department of Clinical Laboratory, Xiangya Hospital, Central South University, Changsha, 410008, Hunan, PR China; 4 Department of Blood transfusion, Xiangya Hospital, Central South University, Changsha, 410008, Hunan, PR China; 5 Department of Emergency Medicine, North Shore University Hospital-New York University School of Medicine, Manhasset, New York, 11030, USA

## Abstract

**Background:**

High-mobility group box 1 (HMGB1) is a late mediator of lethal systemic inflammation. Acute liver failure (ALF) has been shown to trigger systemic inflammation in clinical and animal studies. To evaluate the possibility of HMGB1 cytoplasmic translocation in ALF, we determined whether HMGB1 is released in hepatocytes and end organ in patients with liver failure/injury.

**Methods:**

HepG2 cell were stimulated with LPS or TNF-α, the increase of HMGB1 extracellularly in the culture medium and intracellularly in various cellular fractions were determined by western blot or immunocytochemistry. To observe sub-cellular location of HMGB1 in hepatocytes, liver specimens were obtained from 6 patients with ALF caused by HBV infection, 10 patients with chronic viral hepatitis B, 6 healthy controls, as well as animals model of ALF by intraperitoneal administration of D-GalN (600 mg/kg) and LPS (0.5 mg/kg).

**Results:**

In HepG2 cell culture, LPS or TNF actively induced HMGB1 cytoplasmic translocation and release in a time- and dose-dependent fashion. In animal model of ALF, cytoplasmic HMGB1 translocation was observed in hepatocyts as early as 3 hours post onset of ALF. In patients with ALF caused by HBV infection, cytoplasmic HMGB1 translocation was similarly observed in some hepatocytes of the liver specimen.

**Conclusions:**

Cytoplasmic HMGB1 translocation may occur during ALF, which may potentially contribute to the pathogenesis of liver inflammatory diseases.

## Background

High mobility group box 1 (HMGB1) is a non-histone nuclear protein ubiquitously expressed in eukaryotes, that exerts distinct functions at different subcellular localizations. Within the nucleus, it plays an important role in the regulation of gene transcription [1]. Upon release by phagocytes and damaged/necrotic cells [2-5], extracellular HMGB1 functions as a damage-associated molecular pattern (DAMP), and contributes to the pathogenesis of various inflammatory diseases [6,7].

HMGB1 exerts its effects through the receptor for advanced glycation end products (RAGE) and a number of the Toll-like family of receptors (TLR2/4) [5]. This leads to activation of endothelial [8,9] and immune cells, and consequent release of multiple proinflammatory cytokines [10]. In animal models of infection or local tissue injury, HMGB1 functions as a critical mediator of systemic or local inflammatory injury [11]. In the clinical setting, elevated serum HMGB1 levels have been described in patients with sepsis [2,12,13], pneumonia [14], acute pancreatitis[15], as well as cerebral and myocardial ischemia[16].

Acute liver failure (ALF) is a rare condition in which rapid deterioration of liver function results in altered mentation and coagulopathy, and even mortality. Unlike the United States and many other countries, the primary cause of ALF in China is viral hepatitis B[17,18], which accounts for 74% of cases in Hong Kong[19]. The pathophysiology of ALF remains poorly understood, and thus, it has become an area of great interest. It has been suggested that ALF can trigger systemic inflammation in human clinical trials [20] and animal studies [21]. Patients with ALF have higher circulating concentrations of proinflammatory cytokines [e.g., tumor necrosis factor (TNF)-α, interleukin (IL)-1β, and IL-6] than healthy subjects or patients with acute hepatitis [22,23]. Recently, HMGB1 has been established as a late mediator of lethal systemic inflammatory disease. By itself, or in conjunction with other proinflammatory cytokines (e.g., IL-1β, IFN-γ and TNF-α), HMGB1 amplifies an inflammatory response by stimulating the release of various proinflammatory cytokines [10,24]. In light of the important role of HMGB1 in inflammatory diseases, we sought to determine whether HMGB1 cytoplasmic translocation occurs in hepatocytes following stimulation with exogenous (e.g., bacterial endotoxin) or endogenous (e.g., TNF) stimuli.

Like other inflammatory cytokines released by the liver during early hepatic injury [25-27], HMGB1 may be similarly released by the liver, thereby triggering/contributing to systemic inflammation. HMGB1 is abundantly expressed in hepatocytes, and predominantly localized in the nucleus of quiescent cells. It was previously unknown whether inflammatory stimuli can induce hepatocytes to actively release HMGB1. Given the huge numbers of hepatocytes in the liver, potential HMGB1 release by hepatocytes could contribute to the pathogenesis of liver failure/injury. Here we demonstrated that hepatocytes can actively release HMGB1 after challenge with exogenous (e.g., LPS) or endogenous (e.g., TNF-α) inflammatory stimuli. Furthermore, HMGB1 cytoplasmic translocation was observed in hepatocytes in the animal model of ALF (induced by D-galactosamine and LPS), as well as in patients with ALF.

## Methods

### Cell culture and stimulation

Human hepatocyte cell line HepG2 was obtained from the American Type Culture Collection *(ATCC, Rockville, MD)*, and cultured in Dulbecco Modified Eagle's medium (DMEM) *(Gibco BRL, Grand Island, NY) *supplemented with 10% fetal bovine serum (FBS), 2 mmol/L glutamine and 100 U/mL penicillin and streptomycin in flasks at 37°C, in a humidified atmosphere of 5% CO_2 _in air. Adherent HepG2 cells in 6 well culture plates were gently washed with, and cultured in serum-reduced OPTI-MEM I medium 8 h before stimulation with different concentrations of LPS *(Sigma Chemical Co.) *or TNF-α *(PromoCell, Heidelberg, Germany) *for various time periods.

### MTT assay

Cells were plated at a density of 10^4 ^cells/well on 96-well plates in 200 μl DMEM. After stimulating with LPS or TNF-α for indicated time periods and concentrations, 20 μl of MTT *(Sigma Chemical Co.) *was added to each well and incubated for 2 h at 37°C. After removing MTT solution, 150 μl dimethylsulphoxide *(Sigma Chemical Co.) *was added to each well. The absorbance was determined using an ELISA reader at a wavelength of 570 nm (test) and 690 nm (reference). The spectrophotometer was calibrated to zero absorbance using culture medium without cells. The relative cell viability (%) related to control wells containing cells and culture medium without treatment was calculated by [A] test/[A] control × 100.

### Preparation of cellular extracts

Cells were harvested and washed twice with cold PBS; nuclear and cytoplasmic extracts were prepared according to the method of Schreiber et al. Briefly, the cell pellet was re-suspended in low salt buffer (10 mM HEPES, pH 7.9, 10 mM KCl, 0.1 mM EDTA, 0.1 mM EGTA, 1 mM DTT, 0.5 mM PMSF, 1% Nonidet P-40). After mixing, the intact nuclei were collected by a quick centrifugation, leaving the cytoplasmic fraction in the supernatant. The nuclear pellet was re-suspended in Nonidet P-40 high salt buffer (20 mM HEPES, pH 7.9, 0.4 M NaCl, 1 mM EDTA, 1 mM EGTA, 1 mM DTT, 1 mM PMSF, 1% Nonidet P-40). After mixing at 4°C for 15 min on a shaking platform, the nuclear extract was centrifuged for 5 min in a microfuge at 4°C, and the supernatant was frozen in aliquots at -80°C. The protein content of the different fractions was determined by a Bradford method.

### HMGB1 Western blotting analysis

Cell-conditioned medium was harvested and filtered through Millex-GP *(Millipore, Bedford, MA) *to remove cell debris and macromolecular complexes. Samples were then concentrated 40-fold with Amicon Ultra-4-10000 NMWL *(Millipore, Bedford, MA) *following the manufacturer's instructions. Proteins in the subcellular fractions or concentrated cell culture supernatants were resolved on 10% SDS-PAGE gel and transferred to a polyvinylidene fluoride membrane. After blocking the membrane at room temperature for 2 h, the membrane was incubated overnight at 4°C with primary antibodies specific for HMGB1 *(kindly provided by Dr. Haichao Wang, North Shore-Long Island Jewish Research Institute, NY, USA)*, a cytoplasmic protein *(β-actin; Santa Cruz Biotechnology) *and a proliferating cell nuclear antigen *(PCNA; BD Biosciences)*, respectively. The membrane was then incubated with horseradish peroxidase-conjugated goat anti-rabbit IgG (1:3000 dilution) for 2 h at room temperature. The signal was visualized with ECL detection reagent and quantitated by densitometry using Quantity One software.

### Fluorescence immunostaining

HepG2 cells were cultured on glass cover slips and stimulated with LPS or TNF-α for 24 h. Subsequently, cells were fixed with 4% formaldehyde for 10 min, and permeabilized with 0.2% Triton-X-100 in PBS. After extensive washing with PBS containing 0.2% BSA, cells were sequentially incubated with anti-affinity-purified rabbit anti-HMGB1 antibodies and goat anti-rabbit secondary antibodies conjugated with fluorescein isothiocyanate *(Sigma Chemical Co.)*, and subsequently counterstained with propidium iodide *(Sigma Chemical Co.)*. Images were captured using a fluorescence microscope *(Nikon, Japan)*.

### Cytokine Ab array

Human cytokine antibody array *(R&D, Minneapolis, MN)*, a high throughput technology which detects 42 cytokines on one membrane, was used to determine the profile of cytokines in the culture medium following the manufacturer's instructions. Briefly, the membranes were sequentially incubated with equal volume of cell-conditioned culture medium, primary biotin-conjugated Ab, and HRP-conjugated streptavidin. After exposing to x-ray film, the relative signal intensity was determined using UN-SCAN-IT Gel 6.1 software with reference to the positive controls on the membrane.

### Animal model of ALF

Female BALB/c mice aged 6-7 weeks with a body weight of 18-20 g *(from the Experimental Animal Center of Xiangya hospital, Central South University, Changsha, China) *were handled and treated in accordance with the strict guiding principles of the National Institution of Health for experimental care and use of animals. Mouse ALF was induced by intraperitoneal injection of D-GalN (800 mg/kg) *(Sigma-Aldrich Co., Ltd) *and LPS (0.04 mg/kg) *(Sigma-Aldrich Co., Ltd) *as previously described [28-30]. Three hours following the onset of ALF, mice were sacrificed to harvest liver tissue for immunohistochemistry and hematoxylin-eosin (HE) staining.

### The liver specimens from ALF patients caused by HBV infection

Liver specimens were obtained from 6 patients (5 males and 1 female; mean age, 32 years) with ALF caused by HBV infection who received liver transplantations. Specimens were also collected from 10 (9 males and 1 female; mean age, 36 years) chronic viral hepatitis B patients without liver failure who received liver transplantations or liver biopsy in Xiangya Hospital, Central South University, during a two-year period (2005.5-2007.5). ***Written informed consent was obtained from all patients before performing liver biopsy or liver surgery. This study was approved by the Ethics Committee of Xiangya Hospital of Central South University.***

The diagnosis of chronic hepatitis B was based on elevated values of serum alanine aminotransferase (ALT) for at least 1 year, as well as the presence of serum hepatitis B virus (HBV) markers by ELISA and/or HBV-DNA by polymerase chain reaction (PCR).

**The criteria for ALF**[17]: (*AASLD Position Paper: The management of acute liver failure, 2005) *include evidence of coagulation abnormality, usually an INR ≥ 1.5, and any degree of mental alteration (encephalopathy) in a patient without preexisting cirrhosis and with an illness of < 26 weeks duration. All these patients had positive results of serum HBV markers tested by ELISA and/or HBV-DNA positive by PCR. As controls, normal liver tissue samples were obtained from 6 patients who had no evidence of HBV and HCV infection (4 males and 2 females; mean age, 42 years). Two of these 6 control patients had cholelithiasis, 2 had hepatic cyst, and the remaining 2 had hepatic carcinoma. All the tissue samples were obtained from the adjacent normal liver, identified by histology, of these 6 patients. For all the patients involved there was no evidence of co-infection with other hepatotropic viruses. Further possible causes of liver damage, such as alcohol, drugs or autoimmune diseases were also excluded. Surgically collected liver specimens were fixed in 4% formaldehyde solution, embedded in paraffin wax, and stained with haematoxylin and eosin.

### Immunohistochemical staining

Four micrometers of tissue sections were de-paraffinized, rehydrated, and treated with an antigen retrieval solution (10 mmol/L sodium citrate buffer, pH 6.0). The sections were incubated with a dilution of 1:500 rabbit anti-HMGB1 antibody *(Abcam, UK) *overnight at 4°C, and then incubated with 1:1000 dilution of biotinylated secondary antibody, followed by avidin-biotin peroxidase complex *(DAKO, Carpinteria, CA) *according to the manufacturer's instructions. Finally, tissue sections were incubated with 3', 3'-diaminobenzidine *(Sigma-Aldrich, Co., Ltd) *until a brown color developed, and then the sections were counterstained with Harris' modified hematoxylin. In negative controls, primary antibodies were omitted. Hepatocytes that have brown staining in nucleus area represent normal location of HMGB1, while hepatocytes with brown staining in both cytoplasmic and nucleus area were defined as cytoplasmic translocation of HMGB1. At least 10 high-power fields were chosen randomly, and >1000 cells were counted for each section. Values represent percentages of hepatocytes with HMGB1 cytoplasmic translocation in all hepatocytes counted in each group.

### Statistical analyses

All continuous data were expressed as the mean ± SD. Comparison between groups was performed using Student's t-test or one way-ANOVA analysis. Statistical significance was defined as a P < 0.05. All statistical analyses were performed using SPSS 13.0 for Windows.

## Results

### Effects of LPS or TNF-α on the release of HMGB1 in HepG2 cells

**Figure 1:**
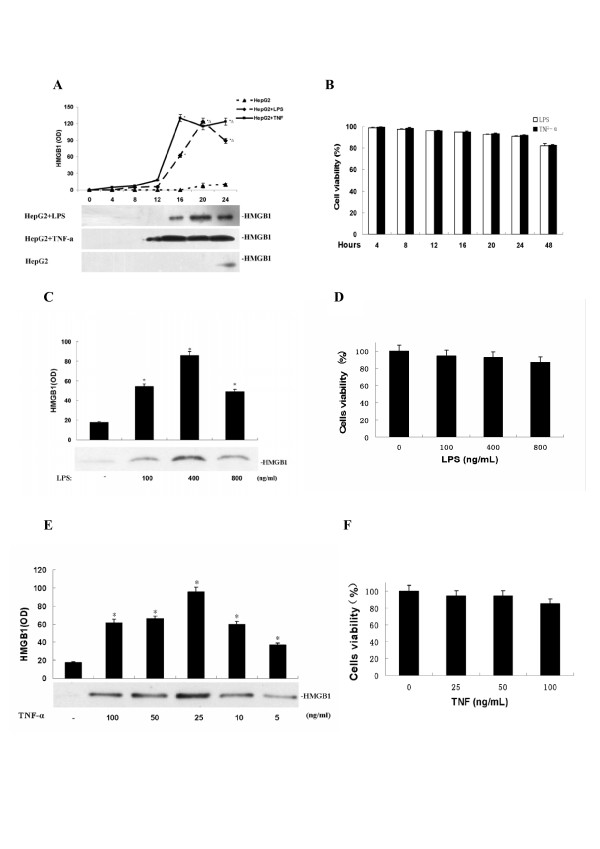
**LPS and TNF-α stimulated HepG2 cells to release HMGB1 in a time- and dose-dependent fashion**. **A, B) **LPS and TNF induced HMGB1 release in a time-dependent fashion. HepG2 cells were stimulated with LPS (200 ng/ml) or TNF-α (25 ng/ml) for indicated time periods, and extracellular HMGB1 levels (Panel A) or cell viability (Panel B) or were determined by MTT or Western blotting analysis, respectively. HMGB1 levels were determined by the relative optical intensity (OD) of the immunoreactive bands on Western blots, and expressed as mean ± SEM of three experiments in duplicate. * *P *< 0.05 versus 0 h treatment group. **D, E, F, G)** LPS and TNF induced HMGB1 release in a dose-dependent manner. HepG2 cells were stimulated with LPS or TNF-α at indicated doses for 24 h cells viability and extracellular HMGB1 levels were determined as described above. *P < 0.05 versus control ("-LPS", Panel C, D; or "-TNF", Panel E, F).

Both exogenous (LPS) and endogenous (TNF-α) inflammatory stimuli induced active HMGB1 release in HepG2 cell cultures in a time-dependent fashion (Figure 1A), starting at 12-16 h post LPS or TNF stimulation. The release of HMGB1 was not dependent on cell death, because the cell viability was not significantly altered by LPS or TNF even at 24 h post treatment (Figure 1B). Furthermore, LPS or TNF stimulated HepG2 cells to released HMGB1 in a dose-dependent manner (Figure 1C and 1E), starting at concentrations as low as 100 ng/ml (for LPS, Figure 1C) or 5 ng/ml (for TNF, Figure 1E), and peaking at concentrations around 400 ng/ml (for LPS, Figure 1C) or 25 ng/ml (for TNF, Figure 1E), respectively. The release of HMGB1 was not dependent on cell death, because LPS or TNF did not significantly affect viability at concentrations (up to 400 ng/ml for LPS, and 50 ng/ml for TNF) that effectively induced HMGB1 release (Figure 1D and 1F). At slight cytotoxic dosages, LPS (800 ng/mL) or TNF-α (100 ng/mL) might also cause passive HMGB1 leakage from necrotic hepatocytes, but the overall extracellular HMGB1 levels were somewhat lower than active release (Figure 1C and 1E).

### LPS and TNF-α induced HMGB1 cytoplasmic translocation in HepG2 cells

**Figure 2:**
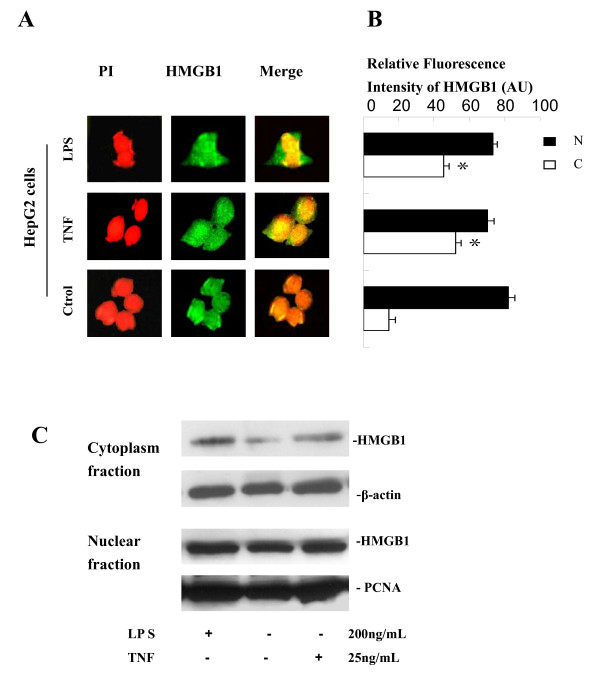
**LPS or TNF-α induced HMGB1 cytoplasmic translocation in HepG2 cells**. HepG2 cells were stimulated with LPS (100 ng/ml in panel A and B, 200 ng/ml in panel C) or TNF-α (25 ng/ml) for 20 h, and monitored for HMGB1 cytoplasmic translocation by immunocytochemistry (Panel A) or by Western blot analysis after cell fractionation (Panel C). **A, B).** HMGB1 immunohistochemistry assay. The relative fluorescence intensity in the nuclear ("N") or cytoplasmic ("C") regions of multiple represent cells were determined using the Image Proplus Software, and expressed as mean ± SEM (in arbitrary units, AU) of three independent experiments. Red: nuclear; green: HMGB1; yellow: merge (original magnification × 400). * *P *< 0.05 versus control. **C).** HMGB1 Western blotting analysis. Following cell fractionation, HMGB1 content in the cytoplasmic ("C") or nuclear ("N") fraction was determined by Western blot analysis.

Following immunostaining, HMGB1 was found predominantly in the nucleus of quiescent HepG2 cells (Figure 2). At 20 h after stimulation with LPS (100 ng/ml) or TNF-α (25 ng/ml), HMGB1 staining was observed in both nuclear and cytoplasmic regions of HepG2 cell culture (Figure 2A and 1B). Consistently, levels of HMGB1 in the cytoplasmic fractions were increased after stimulation with LPS (200 ng/ml) or TNF-α (25 ng/ml) as detected by Western blotting analysis (Figure 2C). Taken together, this experimental data suggests that both endogenous and exogenous inflammatory stimuli can induce HMGB1 nuclear-cytoplasmic translocation in hepatocytes.

### Effects of LPS or TNF-α on cytokine release in HepG2 cells

**Figure 3:**
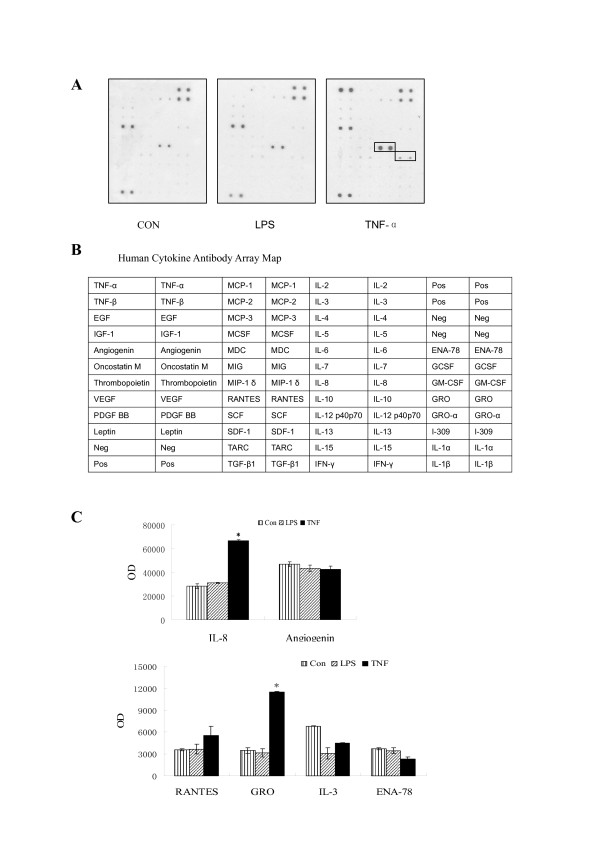
**Effects of LPS or TNF-α on release of cytokines from HepG2 cells**. HepG2 cells were stimulated with LPS (200 ng/mL) or TNF-α (25 ng/mL) for 24 h, and extracellular levels of 42 different cytokines were determined by using human cytokine antibody array. **A, B).** Representative cytokine antibody array. Note that TNF, but not LPS, markedly induced the release of IL-8 and GRO. **C).** Quantitation of relative levels of IL-8 and GRO. The relative levels of IL-8 and GRO were determined using the UN-SCAN-IT Gel 6.1 software, and the optical density (OD) was expressed as mean ± SEM of two different experiment. * *P *< 0.05 versus control group.

Human cytokine antibody array was used to evaluate the release of 42 different cytokines from HepG2 cells at 24 h post stimulation with LPS (200 ng/mL) or TNF-α (25 ng/mL). Interestingly, TNF-α induced the release of two chemokines (IL-8 and GRO) in HepG2 cell cultures, indicating that TNF-α can simultaneously stimulate hepatocytes to release HMGB1 and a subset of chemokines (Figure 3A, B and 3C).

### HMGB1 cytoplasmic translocation in hepatocytes of ALF patients caused by hepatitis B

**Figure 4:**
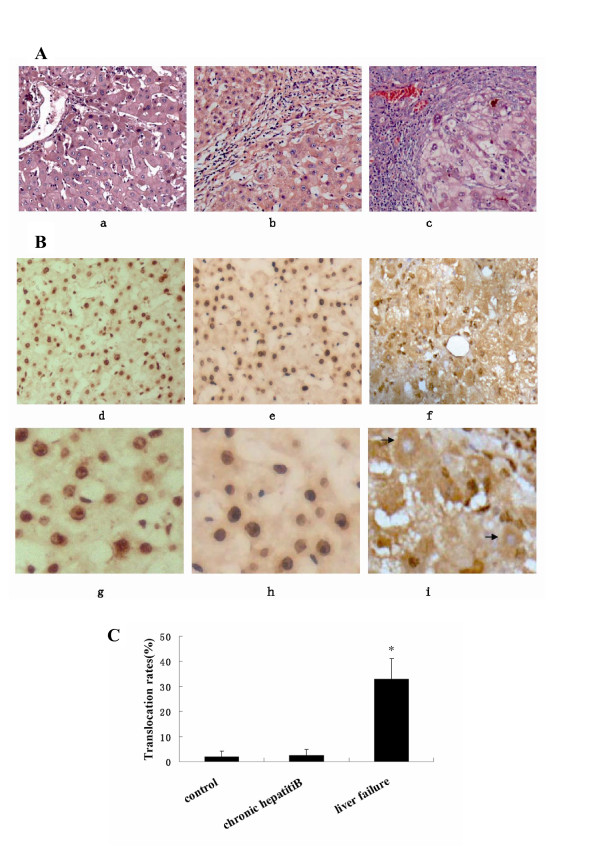
**HMGB1 cytoplasmic translocation in hepatocytes of ALF patients caused by HBV infection**. **A).** HE staining of liver sections (×*200). *a, normal adults; b, chronic viral hepatitis B patients; c, ALF patients caused by hepatitis B (panel c, × 200). **B).** HMGB1 immunohistochemistry. d and g: healthy controls; e and h: chronic viral hepatitis B patients, f and i: ALF patients. d, e and f × 200; g, h and i × 800). Brown: HMGB1; Blue: counterstaining of nucleus with hematoxylin; Arrow: cytoplasmic HMGB1 staining in hepatocytes. **C).** Percentage of hepatocytes with HMGB1 cytoplasmic translocation.*, *P *< 0.05 versus controls or chronic hepatitis B patients.

The pathological findings in the liver of patients with ALF included massive or sub-massive necrosis of liver cells and infiltration of immune cells around the necrotic foci. As a feature of regeneration, enlarged hepatocytes with clear cytoplasm and nucleus were found within the regeneration foci, (Figure 4A). Subcellular distribution of HMGB1 was observed in hepatocytes within the regeneration foci. Interestingly, HMGB1 staining was found in both cytoplasm and nucleus of many hepatocytes in patients with ALF (Figure 4B). In some hepatocytes, HMGB1 was only found in the cytoplasm, and barely detectable in the nucleus, (Figure 4B), suggesting nuclear-cytoplasmic HMGB1 translocation occurs in hepatocytes of patients with ALF. In normal healthy controls or patients with chronic HBV infection but not liver failure, HMGB1 staining was mostly localized in the nucleus, with few cells occasionally positive for cytoplasmic HMGB1 (Figure 4C). In ALF patients caused by hepatitis B, the HMGB1 translocation rates were around 32.84% ± 7.13%, which was significantly higher than that in chronic hepatitis B patients and controls.

### HMGB1 cytoplasmic translocation in hepatocytes of mice with ALF

**Figure 5:**
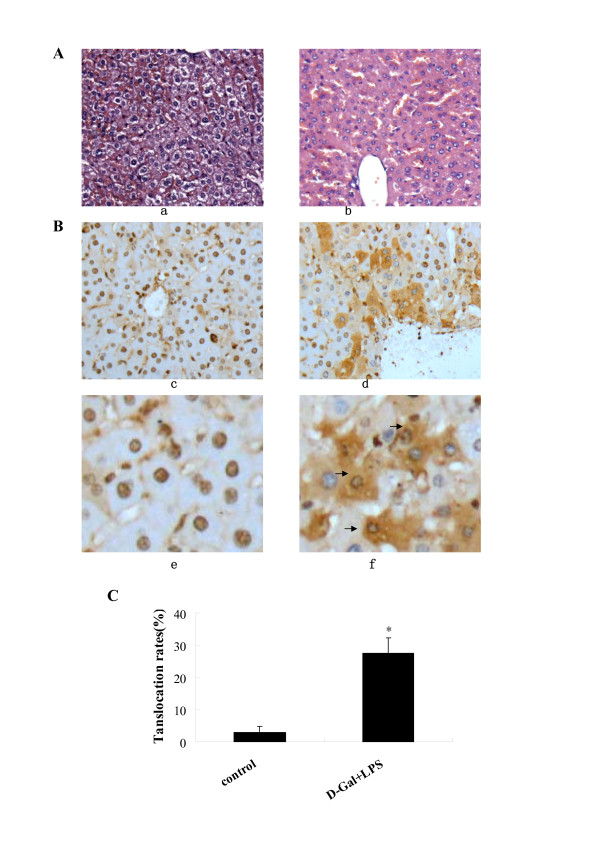
**HMGB1 cytoplasmic translocation in hepatocytes of ALF mice**. **A):** HE staining of liver sections. a: control group; b: D-GalN and LPS treated group. a, b × 200. **B).** HMGB1 immunohistochemistry.c and e: control group; d and f: D-GalN and LPS treated group. c, d × 200; e, f × 800, Brown: HMGB1; Blue: counterstaining of nucleus with hematoxylin; Arrow: HMGB1 cytoplasmic translocation in hepatocytes. **C).** Percentages of hepatocytes with cytoplasmic translocation.* P < 0.05 versus controls.

The pathological findings of liver after exposure to D-Gal and LPS for 3 h showed minor derangement of hepatic plate, and appearance of ballooning degeneration in several hepatocytes. Liver specimens from normal control mice revealed a nuclear localization of HMGB1 in most hepatocytes. Occasionally, HMGB1 staining was found in the cytoplasm of some hepatocytes of control mice (Figure 5B). In contrast, HMGB1 cytoplasmic staining was easily observed in hepatocytes as early as 3 h after administration of D-Gal and LPS. HMGB1 staining was found in both cytoplasm and nucleus of many hepatocytes in the lobes(Figure 5B). In some hepatocytes, HMGB1 was only found in the cytoplasm, showing different stages of HMGB1 cytoplasmic translocation. Consistently, the percentage of hepatocytes with HMGB1 cytoplasmic staining was significantly higher (27.42% ± 4.99%) in the D-Gal and LPS treated group than that in control groups (Figure 5C).

## Discussion

Although etiologies of ALF vary between Western countries and the Eastern developing world, the resulting clinical manifestation is remarkably similar. This reflects common patterns of innate immune responses to various pathogenic factors, such as bacteria toxins, cytokines, and free radicals [31]. Among many others, proinflammatory cytokines (such as TNF-α, IL-1β, and IL-6) may play a common role in the pathophysiology of ALF.

HMGB1 is a nonclassical proinflammatory mediator that is secreted by, and activates proinflammatory responses in, phagocytes and endothelial cells [5]. To appreciate a potential role for HMGB1 in ALF, we investigated whether HMGB1 can be released by hepatocytes in the liver of patients or animals with acute liver failure/injury.

At nontoxic concentrations, both exogenous (e.g., LPS) and endogenous (TNF) inflammatory stimuli induced HMGB1 nuclear-cytoplasmic translocation, and subsequent release in human hepatocyte HepG2 cells. In 1999, Wang et al first reported that monocytes/macrophages actively release HMGB1 in response to exogenous (e.g., LPS) or endogenous inflammatory stimuli (such as TNF-α, IL-1β, or IFN-γ) [2]. Subsequently, active HMGB1 release has been shown in non-immune cells such as pituicytes and enterocytes [32,33]. In the present study, we found that hepatocytes similarly translocate nuclear HMGB1 to cytoplasm, and release it following LPS or TNF-α stimulation. The active release of HMGB1 was not dependent on cell death, as other chemokines (such as IL-8 and GRO) were similarly released by hepatocyte following TNF stimulation.

Hepatocytes are responsible for multiple functions, including regulation of homeostasis, blood sugar, metabolisms of lipids and amino acids, bile formation, and detoxifying capacities. Our present study raises the possibility that activated hepatocytes could be a source of extracellular HMGB1, which may contribute to inflammatory response during ALF[34]. Trying to understand if hepatocytes can release other cytokines besides HMGB1 when stimulated with LPS or TNF, human cytokine antibody array was employed to test 42 typical cytokines in the supernatant. Interestingly, both LPS and TNF-α stimulation failed to induce significant release of typical inflammatory cytokines, such as IL-1, TNF, IL-6, IFN-γ, in HepG2 cells. Nevertheless, a few chemokines (e.g., IL-8 and GRO) were released by hepatocyte following TNF stimulation but its relevance to HMGB1 release is a subject of on-going investigation.

In China, a large proportion of ALF are caused by HBV infection [35], thus we evaluated HMGB1 cytoplasmic translocation in ALF patients caused by hepatitis B. Consistent with previous reports, we found histopathological changes; these included massive, sub-massive or bridging necrosis with immune cell infiltration and regeneration nodular of hepatocytes in liver sections of patients with ALF. Interestingly, HMGB1 cytoplasmic translocation is clearly observed in regenerated hepatocytes of patients with ALF caused by HBV infection, but not in patients with chronic HBV infection. One limitation of the clinical study is that liver tissue samples were obtained during liver transplantation surgery, and thus did not represent early clinical manifestation immediately after the onset of ALF. Consequently, we employed a murine model of ALF induced by co-administration of D-GalN and LPS to further investigate the translocation of HMGB1 in hepatocytes during liver failure/injury. In this model of ALF, liver injury is dependent on the induction of proinflammatory cytokines (such as TNF and IFN-γ) [28,36], and loss of liver function and hepatic histology occur typically 6-12 h post administration of D-Gal and LPS [37]. In the present study, we found that HMGB1 nuclear-cytoplasmic translocation occurs as early as 3 h after injection of D-GalN (600 mg/kg) and LPS (0.5 mg/kg). At this early time point, there was no significant necrosis of hepatocytes and the rate of hepatic apoptosis (detected by TUNEL assay) was still low (<10%), but the percentage of hepatocytes with HMGB1 cytoplasmic translocation was already rather high (27.42% ± 4.99%).

HMGB1 can bind to several potential receptors (e.g., RAGE and TLR2/4) and that may be highly expressed during inflammation or in primary hepatocelullar carcinoma. We can't exclude the possibility that the HMGB1 observed in cytoplasm of hepatocytes in ALF patients was HMGB1 from the nucleus of necrotic cells that combined with a receptor. This possibility is unlikely, because LPS- or TNF-α-induced cytoplasmic HMGB1 translocation was observed in hepatocytes in the absence of cell death in vitro. To examine HMGB1 translocation in vivo, we strategically chose a very early sampling time (after onset of acute liver injury/failure), when necrosis of hepatocytes was rare but the cytoplasmic HMGB1 was easily seen.

It has been widely suggested that re-localization and accumulation of HMGB1 in the cytoplasm is a necessary step for its extracellular release, raising the possibility that hepatocytes might be a source for extracellular HMGB1 during ALF. It is reasonable to propose that during liver injury/failure HMGB1 is released as a danger signal from activated or damaged hepatocytes as well as immune cells and necrotic cells. Collectively, extracellular HMGB1 itself, or in conjunction with other inflammatory stimuli, orchestrates a rigorous inflammatory response.

To our knowledge, this is the first study to link hepatic HMGB1 release and potential pathophysiology/prognosis of ALF. Indeed, circulating HMGB1 levels were found to be elevated in patients suffering from liver failure caused by hepatitis B (data not shown). The source of circulating HMGB1 may include activated innate immune cells or non-immune cells (such as hepatocytes).

## Conclusions

Taken together, our in vitro experiments revealed that active nuclear-cytoplasmic HMGB1 translocation and release occurred in HepG2 cells upon stimulation with both exogenous (LPS) or endogenous (TNF-α) inflammatory stimuli. Furthermore, cytoplasmic HMGB1 was observed in hepatocytes of patients with ALF caused by HBV infection, as well as in murine models of ALF. Together, these observations raised important questions regarding potential pathogenic roles of HMGB1 in ALF or liver injury, which warrants further investigation in future studies.

## Abbreviations

HMGB1: High-mobility group box1; ALF: acute liver failure; DAMP: damage-associated molecular pattern; RAGE: receptor for advanced glycation end products; TNF: tumor necrosis factor; IL: interleukin; IFN: interferon; HBV: hepatitis B virus; D-GalN: D-galactosamine.

## Competing interests

The authors declare that they have no competing interests.

## Authors' contributions

XGF and HCW conceived and supervised the project; RRZ, SSZ and MXZ designed the experiments; RRZ, PZ, BXZ,XHD and HBL performed the experiments and collected data; SSZ, MXZ and NL analyzed data; RRZ and HCW wrote the manuscript. All authors read and approved the final manuscript.

## Pre-publication history

The pre-publication history for this paper can be accessed here:

http://www.biomedcentral.com/1471-230X/11/21/prepub
